# Gender Differences in Older Adult Social Participation

**DOI:** 10.1093/geroni/igaf122.3453

**Published:** 2025-12-31

**Authors:** Eric Vogelsang, Sara Moorman

**Affiliations:** California State University, San Bernardino, San Bernardino, California, United States; Boston College, Chestnut Hill, Massachusetts, United States

## Abstract

A growing body of research has found that older adult social participation may benefit various aspects of health and well being. While some of this work has found these associations differ by gender, there has been little rigorous investigation into how social participation itself varies between older men and women. Using 10 waves of the Health and Retirement Study, we study how (a) the breadth of social activities, (b) social activity frequency, and (c) the social determinants of social participation differ by gender. Generally, we find that men and women were involved in approximately the same number of social activities and at the same total level of activity frequency. However, gendered differences in population composition (e.g., educational attainment, marital status) suppress greater social participation for women. After controlling for these compositional differences, we find that women had slightly greater participation scores—mostly attributable to volunteering and religious service attendance. We also found that social activity decline begins at about age 65 for men, but age 70 for women. When investigating the social determinants of social participation, our results indicate that men suffer greater “penalties” for being divorced or never married. In addition, we found that increased social participation among those currently working only applied to men. Last, we found that the positive associations between high socioeconomic status and social participation was much stronger in magnitude for women. These results demonstrate how gendered social norms and inequality may impact this important aspect and correlate of “healthy aging.”

